# SWOT analysis of the models used by social enterprises in scaling effective refractive error coverage to achieve the 2030 in SIGHT in Kenya

**DOI:** 10.1038/s41598-024-54493-z

**Published:** 2024-02-14

**Authors:** Shadrack Muma, Kovin Shunmugam Naidoo, Rekha Hansraj

**Affiliations:** 1 Department of Optometry, College of Health Sciences, University of KwaZulu-Natal, Durban, South Africa; 2 OneSight EssilorLuxottica Foundation, Paris, France

**Keywords:** Telemedicine, Social enterprises, Refractive error, Uncorrected refractive error, Effective refractive error coverage, 2030 IN SIGHT, Strategic plans, Health care, Medical research

## Abstract

Uncorrected refractive error has predominantly been delivered through commercial entrepreneurship in Kenya. However, to achieve the 2030 IN SIGHT, integration of other forms of entrepreneurship such as the social entrepreneurship is desirable to supplement the efforts of the dominant commercial entrepreneurship. Therefore, this study intended to undertake a SWOT analysis of the current models used by social enterprises in scaling effective refractive error coverage to achieve the 2030 IN SIGHT in Kenya. A review of the seven national strategic plans for eye health in Kenya was undertaken to get a glimpse on the efforts directed towards uncorrected refractive error in achieving the 2030 IN SIGHT. The review was inclined towards assessing the efforts directed by the strategic plans towards scaling human resource, spectacle provision and refraction points. A SWOT analysis was undertaken based on the financial, impact and the approach report for each model. A key informant interview was conducted with a representative and three to five members of the social enterprise about the model. Thereafter, the modified SWOT analysis based on the review and the interview was presented to the representatives of the social enterprises. Purposive sampling was used to identify seven models used by social enterprises in the delivery of refractive error services in Kenya. Finally, the recommendations were presented to key opinion leaders for an input through a Delphi technique. Out of the seven national strategic plans for eye health reviewed, only the strategic plan 2020–2025 intends to establish optical units within 15 different counties in Kenya. Of the seven models currently utilized by social enterprises, only the Kenya Society for the Blind has integrated the telemedicine concept. On application of mHealth, all of the social enterprises models tend to embrace the approach for screening activities. None of the models has a strengthened referral pathway utilizing telereferral and telemedicine. Out of all the models, only Operation Eyesight Universal, Fred Hollow Foundation and Peek Acuity do not depend on sales of subsidized spectacles for sustainability. Every model has the capacity to propel the delivery of refractive error services depending on its comprehensiveness. However, for the 2030 IN SIGHT to be achieved, models prioritizing human resource through telemedicine integration, service provision across all sectors, awareness creation and enhancing cost efficiency are desirable.

## Introduction

Uncorrected refractive error (URE) is the presenting visual acuity of less than 6/12 in the better eye with an improvement of at least two lines after refraction^1^. According to the Global Burden of Disease 2020, 338.8 million people globally had moderate and severe visual impairment (MSVI) with a projection of 535 million by 2050 if no interventions are taken to reduce avoidable blindness^2^. The 2030 in SIGHT demands that by 2030, no-one should experiences unnecessary or preventable sight loss and eye care and rehabilitation services should be accessible, inclusive and affordable to everyone, everywhere, whenever they are needed^3^. A study by Jung^4^ showed that a significant unmet healthcare needs have been observed among poor people living in remote areas. However, a social enterprise (SE) which is an organization which participates in business ventures through a commercial approach in order to fulfill a social purpose^5^, could potentially enhance the health and wellbeing of vulnerable groups through provision of affordable healthcare products and services^6^. Notwithstanding, social enterprises (SEs) have been shown to engage in various programs including provision of cost effective services and products to population more likely to suffer from health inequalities^7,8^. Even though in Kenya the commercial enterprises which majorly prioritize profit generation dominates the optical industry^9^, warranting the need for evaluating the contributions of different sectors including the SEs towards achieving the 2030 in SIGHT. Therefore, this study intended to assess the strength, weaknesses, opportunities and threats (SWOT) of the current models used by SEs, in scaling effective RE coverage.

While, since the late 1990s, the concept of social entrepreneurship has been used in different countries all over the world as a viable tool in delivering social goals, developing policies to recognize social entrepreneurship as a means of providing public services to meet the changing needs in a society is still lacking^10^. The existence of SE has been attributed to the market failure and a social value for the public good^11^. Social enterprises tend to bridge human resource gap in the eye health ecosystem in developing countries in Kenya through approaches such as skills development^12^. While in Kenya, conventional training of human resource supersede skills development, limited resources hinders the government from training enough eye care professionals to attend to the growing population. However, with approximately 15.5% of the Kenyan population being in need of eye care services^13^, cost effective approaches such as the skills development undertaken by SEs could be integrated within the existing workforce. With emergence of innovative approaches such as telemedicine which is the provision of health care remotely via information and communications technology^14^, integration is desirable within the existing models used by SEs. Based on socioeconomic perspective, telemedicine has contributed to providing healthcare to previously underserved regions with provision of care which was not previously deliverable^15^. With the minimal information on the innovative components utilized by different SEs models, this study intended to identify what makes SEs models unique and ideal in scaling effective RE coverage.

The increased interest in SEs is as a result of the role they play in addressing unresolved social problems on an international scale while enhancing human development around the world and improving quality of life^16^. This implies that the involvement of SEs in improving health and wellbeing represent a shift on the potential role for business in promoting health beyond profit^17^. Although SEs experiences challenges around sustainability just like any other business, the innovative nature makes SEs to thrive. Social enterprises have been shown to respond to societal challenges through social innovation and making sure that the solution is accessible based on a viable business attention^6^. However, minimal information exists on how the existing SEs models in Kenya enhance sustainability. In Scotland, SEs have been shown to improve health outcomes through direct delivery of primary healthcare and community development program addressing such vulnerabilities^18^. Even though aspects around community engagement, legal compliance, accessibility, affordability, acceptability and the quality of services delivered by SEs remains unknown in Kenya, such information is desirable. In developed countries such as United Kingdom, there exist specific legal entities for SEs which encourages SEs to form frameworks^12^. Therefore, this study intended to evaluate the current models used by SEs with an aim of providing recommendations inclined towards scaling effective refractive error (RE) coverage in Kenya.

## Materials and methods

This study was conducted in two phases. The first phase entailed a scoping review of the seven national eye health strategic plans for eye health in Kenya^13,19^. The review was anchored on aspects around human resources; refraction points/vision centres, refractive error and spectacles provision. Google Scholar was the main search engines used to extract information regarding the existing national strategic plans for eye health in Kenya. Additional information was retrieved from the Ministry of Health website and policy documents on eye health in Kenya. A Boolean operator of “AND” and “OR” was used. The keywords used were as follows: vision centres OR refractive error OR eye health OR refraction OR national strategic plan AND Kenya. This review was intended to understand the government of Kenya efforts towards addressing URE and the recommendations in the strategic plans. The seven national strategic plans for eye health review were downloaded and thereafter reviewed systematically with a focus on the recommendations on addressing URE from the first strategic plan and the achievements in the subsequent strategic plan. The intention of the review was to provide a justification for the need of a specific strategic plan for URE in Kenya. The proposed strategic plan for URE was presented to representatives from SEs and key opinion leaders for their input.

Seven SEs delivering RE services in Kenya were purposively identified. The SEs included the Dot glasses, Operation Eyesight Universal, Fred Hollow Foundation, Eye Rafiki, Peek Acuity, The Kenya Society for the Blind and VisionSpring. The rationale for choosing the seven SE was based on their distinct models replicated in different parts of the world to deliver RE services. The contact details for the SEs were retrieved from their official websites. The SEs were contacted through emails and telephonic calls with an overview of what the study was about. The SEs were asked to endorse a representative with understanding on the model used to participate in the study. The condition for one to be included as a representative to participate in the study, one ought to have engaged in the implementation of a RE program within the SE. The models used by the SE were explored through a telephonic interview with representatives provided by the SEs. The interviews entailed an inquiry of the approaches they are applying in the delivery of RE services, quality and cost of care, acceptability, accessibility, affordability of their products to the base of pyramid population, community engagement and the challenges experienced with the delivery approaches including sustainability. The samples of questions used to seek information from representatives of SEs about the model are shown in Table 1.

**Table 1:** Sample of questions presented to representatives of social enterprises and the key targeted indicators.

Questions	Indicators for the 2030 IN SIGHT
1. Could you provide a brief overview of the current model your organization is using to deliver refractive error services?	Accessibility, availability, awareness and affordability
2. What do you think makes this model fit to achieve the 2030 IN SIGHT?	Accessibility, availability and affordability
3. Do think there are certain challenges with the current model your organization is using when it comes to achievement of the 2030 IN SIGHT?	Inability to enhance accessibility, affordability and availability
4. Do you think a modification to the model is desirable?	Flexibility

During phase two, a SWOT analysis was undertaken to assess the internal and external factors for each model. A review of the existing documents such as the financial reports, impact reports and reports around the approach was undertaken. PubMed and Google Scholar were the main search engines used to extract information regarding the financial reports, impact reports and reports around the approaches used by the seven SEs in Kenya. Additional information was retrieved from the SEs websites. A Boolean operator of “AND” and “OR” was used. The keywords used were as follows: financial report OR model approach OR refractive error OR impact report OR SEs AND Kenya. The rationale for the review was to obtain an objective SWOT of the models given that most organizations may not provide the weaknesses of their approaches to an external researcher. The second step entailed an online key informant interview with three to five members of each SE. The key informant interview comprised of open ended questions adopted from a study by Zoschke et al.^20^ as shown in Table 2. After the key informant interview with all the seven SEs, the responses were crosschecked with the SWOT analysis from the review. Additional information retrieved from the key informant interviews was included in the SWOT analysis from the review. Thereafter, the final SWOT analysis was presented to the three to five representatives from SEs for their view on the relevancy of the proposed components within the existing models.

**Table 2:** The SWOT analysis questions.

Strengths:
1. What are the organization's advantages?
2. What can you do better than others?
3. What unique or lowest-cost services can you provide patients?
4. What do patients in your market see as your organization’s strength?
Weaknesses:
1. Upon what factors could the organization improve?
2. What are patients in your market likely to see as your organization's weakness?
3. What lack of services loses your organization patients?
Opportunities:
1. What good opportunities are available to your organization?
2. What are the new and exciting trends your organization can try?
3. What new changes to governmental regulation/policy can benefit your organization?
Threats:
1. What problems does your organization face?
2. Of what are your organization's competitors taking advantage?
3. Do evolving technologies and new services threatening your organization's position in the minds of patients?
4. Does your facility have cash-flow problems?
5. Could any of your weaknesses threaten quality patient care?

Finally, the proposed aspect to be integrated into the existing models used by SEs was presented to ten key opinion leaders through a Delphi technique. The rationale was to get the broader picture of whether the proposed revisions within the existing models would impact on effective RE coverage and the 2030 IN SIGHT in Kenya. The conditions for inclusion as a key opinion leader was that one had to have been involved in the social entrepreneurship and clinical experience on RE, advocating for the relevance of the concept of social entrepreneurship integration into the eye health ecosystem in Kenya and influence on policies around the eye health in Kenya. The key opinion leaders comprised of an administrator in-charge of eye care services at the ophthalmic service unit Kenya, an ophthalmologist representing the ophthalmological society of Kenya, an optometrist representing the optometrists association of Kenya, an Information and Communication Technology expert from an international SE, an optometrist in-charge of training of the optical technicians, a policy expert representative from the Kenya Society of the Blind, the head of partnership wider NGOs Africa from an international SE, two ophthalmologists operating regional SE and an ophthalmic clinical officer representative.

### Key opinion leader’s selection, recruitment and retention

Within our expert panel, we determined that three Delphi rounds using email for correspondence would be sufficient to achieve consensus and stability^21,22^. We aimed to retain a minimum of 10 key opinion leaders after three rounds of Delphi participation, and based on our experience with previous Delphi studies, we planned for 40% attrition in each round. To ensure that we achieve the minimum number of 10 key opinion leaders, we estimated that 35 invitees would be required in the first round of the Delphi. We adopted two approaches to convene the key opinion leaders engaged in eye care delivery in Kenya. Rather than approaching the ophthalmic service unit Kenya alone which the Ministry of Health organ coordinating eye care services, we chose a group of stakeholders in eye health working towards achieving universal health coverage to contribute towards validation of the proposed approaches to be integrated into the existing models. Second, key opinion leaders in our initial set of 35 contacts were invited to recommend colleagues who, in their opinion, might be interested in participating in this Delphi on the basis of their work or expertise. Throughout the Delphi process, key opinion leaders were blinded to the identity of the others, except for the individuals who referred us to subsequent key opinion leaders. Survey content was never associated with a key opinion leader identifier; only the researchers could associate key opinion leaders with responses. All the included questions from the survey concerned the respondents’ area of professional expertise. An email reminder was sent to key opinion leaders on a weekly basis to increase the response rate.

### Delphi rounds, data collection and analysis

A systematic and meaningful synthesis of responses was ensured through drafting and refining the questions asked of the key opinion leaders in every round. The questionnaire was piloted among members of our authorship team who were not directly involved in designing the Delphi. We communicated with the key opinion leaders in English and used Google Forms to conduct our surveys.

A pre-specified definition of consensus was developed based on two criteria^23^. First, the key opinion leader was eligible to have achieved consensus around a given survey item if at least 70% of respondents agreed with that item. When using a five-point Likert scale, we defined “disagreement” as a score of two or less. This criterion ensured that a strong majority of respondents agreed with any included survey item. Second, an item was said to have achieved consensus only if none of the dissenting respondents raised concerns that were fundamentally incompatible with the inclusion of that survey item.

This criterion aligns with approaches from formal consensus decision-making, where a structured discussion is used to understand and resolve the merits and drawbacks of a given proposal^24^. This approach recognizes that essential insights can be tendered by a minority of decision-makers, and attends to the substance of minority opinions. Procedurally, these minority opinions were gathered by requiring that key opinion leaders offer free-text comments if they disapproved of a survey item. We analysed these free-text responses and incorporated that feedback into subsequent rounds of the Delphi and into the final task shifting framework. As the analysis advanced, an emphasis on and reiteration of certain issues above others became more apparent. These elements coalesced into the final categories and themes.

Round I was designed to elicit broad and general concepts from the key opinion leaders using unstructured, open-ended, questions:

1. What is, in your opinion, the potential of the proposed approaches for integration into the existing SEs models and effective RE coverage?

2. What are the three to five characteristics of refractive error that make it amenable to modification of the current models used by SEs in Kenya?

3. What are three to five examples of activities that the Ministry of Health could undertake to ensure success of the SEs models?

Following Round I, the researchers combined and analysed the key opinion leader’s responses in taxonomy according to common themes and categories. We attempted to make the items on each list mutually exclusive and comprehensive. We synthesized these findings in a survey to elicit participants’ level of agreement with each of the themes and categories on a five-point Likert scale for Round II. This survey also offered free-text response options for key opinion leaders to add additional comments or categories as required.

Once we received all responses from Round II, these data were again reviewed by the researchers. Concepts were eliminated and retained on the basis of the key opinion leader’s scores and collapsed into more general categories, including a definition of task shifting and telemedicine, the purpose of task shifting ad telemedicine, opportunities arising from task shifting and telemedicine programmes, and conditions required for the implementation of task shifting and telemedicine. These results were sent back to the key opinion leaders as a survey for Round III. Key opinion leaders were asked to review the final list of items, state whether they agreed or disagreed with each item, and voice concerns or comments in free text. Following Round III, the researchers integrated the experts’ consensus responses into a reasonable and manageable set of concepts and sub-concepts to form the framework^23^.

The statistical data analysis for quantitative data was conducted in the Statistical Package for the Social Sciences version 29.0.0, 2022. The quantitative data was analysed through descriptive statistics of frequencies and percentages. The qualitative data was analyzed thematically using NVivo Software, Version 11. Thematic analysis was carried out by categorizing the codes into categories using NVivo Software, Version 11 and themes based on the semantic meaning of the codes. It was an iterative process consisting of both deductive and inductive processes^25^. Initial codes and categories were generated from the interview guides (deductive process). New categories that consist of similar codes were added as required to capture the participants’ comments in details (inductive process). During this inductive process, the themes were identified by repetitions (the more the concept appears in the text, the more likely it is to be a theme), similarities and differences^26^.

### Ethics approval and consent to participate

This study was performed in accordance with the Declaration of Helsinki, and has been approved by the Biomedical Research Ethics Committee (BREC/00004105/2022) and Maseno University Ethics Review Committee (MUERC/1051/22). Informed consent to participate in the study was obtained from all participants.

## Results

### Demographics of the participants

All of the representatives from the seven SEs whose models were included in this review, responded to the interview. The representatives constituted (n = 4; 57%) females and (n = 3; 43%) males. The key opinion leaders comprised of (n = 4; 40%) females and (n = 6; 60%) males.

### Phase one

#### Review of the national strategic plans for uncorrected refractive error in Kenya and recommendations

**Figure 1:**
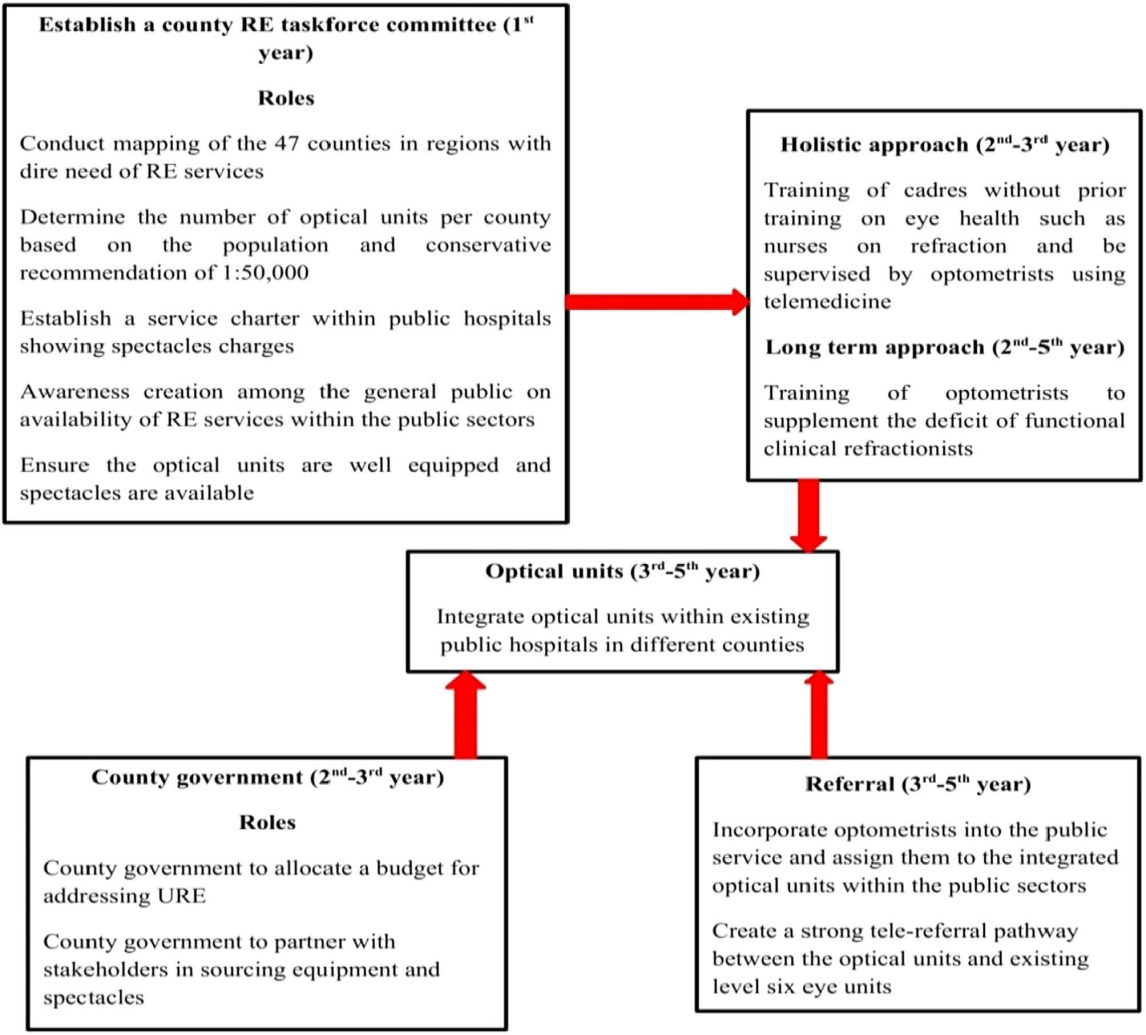
Proposed national strategic plan for uncorrected refractive error in Kenya.

According to the Kenya Ministry of Health^13^, an estimated 15.5% of Kenyans are in need of eye care services. As a result, seven National Eye Health Strategic Plans have been developed with different themes to address eye health in Kenya. However, a critical review of the strategic plans denoted that minimal attention has been directed towards addressing URE in Kenya. For instance, only the national strategic plans for the periods 2012–2018 and 2020–2025 provide recommendations on URE, while the other five national strategic plans only define URE and denote that the prevalence URE remains unknown in Kenya. Notwithstanding, the strategic plans like that for the period 2012–2018^19^ denoted the importance of training human resource to undertake refraction and the establishment of optical units at secondary and tertiary levels. However, as at 2018, the evaluation report showed that the majority of the public eye units were not well equipped with most units having only slit lamps and ophthalmoscopes but lacking retinoscopes and trial boxes^13^. This is a clear indication that URE receives minimal attention in the public health sector. In the review of the national eye health strategic plan 2020–2025, an intention to establish optical units in 15 counties has been proposed within the strategic plan. However, the plan does not show how they will cost effectively scale human resource to be deployed in the suggested optical units. Therefore, with the burden of URE, a national strategic plan for addressing URE was proposed as shown in Fig. 1.

All of the key opinion leaders (100%) agreed that the proposed approach is relevant in addressing URE since minimal attention has been directed towards URE in Kenya. The key opinion leaders argued that designing a specific URE strategic plan will motivate the government to act and allocate resources towards addressing URE (quotes 1–2).

1. I will say that with lack of population based studies, minimal attention has been directed towards addressing refractive error, as a result, I think adopting this specific framework more inclined towards addressing refractive error will ensure that attention is directed towards addressing this challenge—Opinion leader#03

2. This framework can really work given that the budget allocation towards addressing uncorrected refractive error is absent in most counties, hence if this approach is adopted then the small allocation will scale service delivery in Kenya—Opinion leader#07

All of the key opinion leaders agreed that the proposed strategic plan for addressing URE in Kenya is ideal as it intends to address the human resource and infrastructure challenges. The key opinion leaders denoted that advocating for scaling refractive error services within the public health sector is crucial as it scales availability (quote 3–4).

3. In my own view I’d like to say that coming up with this plan which targets human resource is worth as scaling services without considering human resource may produce minimal impact—Opinion leader#03

4. Talking about telemedicine and a strengthened referral pathway is crucial for refractive error service delivery and if the public health sector can result to a scaled refractive error service delivery—Opinion leader#06

All of the SEs representatives reported that the proposed national strategic plan for URE is suitable but the task force committee should include different sectors (quote 5).

5. I will confidently say that we need something of this sort more specific towards addressing uncorrected refractive error in Kenya. however, the taskforce committee should constitute individuals from different sectors with partnership being key—Social enterprise representative#02

### Phase two

#### A SWOT analysis of the current models

##### Eye Rafiki model

Table 3 and 4 details the SWOT analysis of the Eye Rafiki model.

**Table 3:** Strength and weaknesses of the eye Rafiki model.

Strengths	Weaknesses
Human resource and training	Human resource and training
Identify community members and training them on the basics of refraction	Lack of a structured approach in recruiting community members for skills development
Engaging the Ministry of Health to establish the scope of practice for individuals with skills development	The established scope of practise by the Ministry of Health limits the trainees from conducting refraction hence contradicting the training being undertaken
Advocated for recognition of the training by the Technical Vocational Education and Training in Kenya	Lack of a structured certification of the trainees
Training on glazing of lenses	Limited training capacity
Difficulty in mobilization of candidates for training	Lack of licensing of the trainees to operate smoothly
Vision centres establishment	Short period of training on refraction
Established vision centres within different remote areas in Kenya	Engaging a strategic partner who undertakes execution roles with minimal understanding on URE and the worth of quality training
In person supervision of the trainees at the vision centres	Vision centres establishment
Equipping the vision centres with the required materials for glazing and undertaking refraction	Allowing the trainees to establish vision centres before acquiring the experience to operate independently
Integration of vision centres within government primary healthcare facilities	Lack of population based studies to guide on the rationale for establishing the vision centres
Community engagement	Infrastructure not appealing to the patients
Undertaking community vision screening	Community engagement
Quality and cost of care	Lack of partnership between the trainees and the local administration officials
Selling subsidized spectacles to the community members	Minimal engagement with the Community Health Volunteers
Sustainability	Quality and cost of care
Generating income from sales of glasses at the vision centres and during vision screening events	Lack of a remote supervision for the trainees
	Sustainability
	Lack of innovative approaches to scale services and enhance awareness on their existence in the remote areas
	Lack of a partnership between the trainees and established optical units
	Weak referral pathway

**Table 4:** Opportunities and threats of the eye Rafiki model.

Opportunities	Threats
Competitive recruitment of community members for skills development	Lack of policy regulation for SEs in Kenya
Advocate for modification of the scope of practice for the trainees to undertake refraction	Unhealthy competition between existing eye care professionals and the trainees
Proper certification of the trainees is desirable	Conflict of interest between existing eye care professionals and the trainees
Integrate training into existing training institutions offering eye health courses in Kenya	
Integrate the trainees as paraprofessionals under an existing regulating body for eye health professionals	
A 6 month training followed by remote supervision through telemedicine is desirable	
Clinical exposure of the trainees within private and public health sector is desirable for the trainees	
Prioritize population based studies	
Strengthen partnership between trainees and existing eye health professionals	
Utilization of mHealth applications to undertake community screening	

The representative from the Eye Rafiki and the team members reported that the SWOT analysis provides the actual situation they are in and agreed that the opportunities are ideal (quote 6).

6. The weaknesses identified from this SWOT analysis are things we experience and we anticipate that implementation of the opportunities identified is worthy—Representative from Eye Rafiki

**Figure 2:**
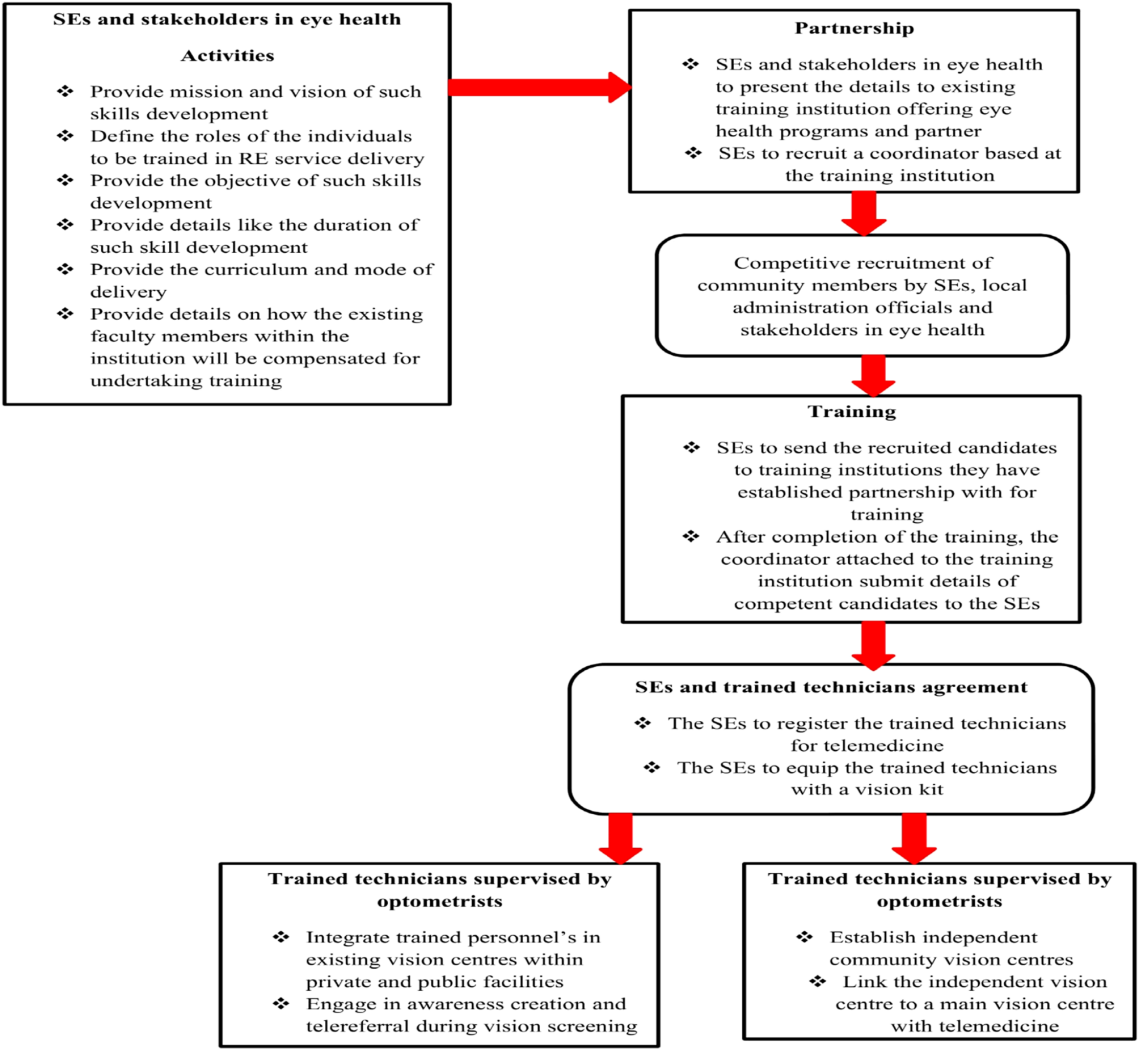
Proposed stages for integrating skills development in existing training institutions offering eye health programs.

Based on the input from the Eye Rafiki representatives, stages for integrating skills development in existing training institutions offering eye health programs was proposed as shown in Fig. 2.

A quarter of the key opinion leaders (n = 7; 70%), agreed that the proposed approach for the integration of skills development into existing training institutions offering eye health programs is a good approach and should be adopted. The key opinion leaders denoted that this approach has the potential to scale human resource in a cost effective way. They also argued that prioritizing telemedicine utilization will scale effective RE coverage in Kenya (quotes 7–8).

7. The interesting part of this proposed approach is that it intends to scale human resource in eye health which I think will scale refractive error service delivery in a cost effective way—Opinion leader#03

8. In Kenya, the eye health sector hasn’t prioritized primary vision technicians but I think if this approach is adopted then service delivery will be scaled as telemedicine is also prioritized—Opinion leader#06

All of the key opinion leaders (100%) agreed that the holistic approach is relevant within the Kenyan context as it remains cost effective when compared to the conventional approach. The opinion leaders denoted that an approach where primary vision technicians can receive support from optometrists through telemedicine can enhance quality service delivery (quotes 9–10).

9. We always have problems with primary vision technicians as we question their quality when it comes to refractive error service deliver, but if they can be supervised by qualified eye care professionals like optometrists through telemedicine then it’s a good approach—Opinion leader#05

10. I think telling primary vision technicians to come back for upgrading is a good think but because resources are limited, it will not be cost effective—Opinion leader#01

All of the key opinion leaders (100%) agreed that the clinical exposure approach is relevant and should be prioritized for quality RE service delivery. The key opinion leaders argued that it will relevant if the primary vision technicians can be integrated into the primary level public and private sectors. However, they stated that it may a long term approach as currently cadres such as optometrists remain outside the public service in Kenya (quotes 11–12).

11. It’s interesting to come across this suggestion of clinical exposure for primary vision technicians as currently the reason as to why many primary vision technicians in Kenya find it hard to operate smoothly is because of questions about the quality they can deliver—Opinion leader#04

12. I will say it will be hard to prioritize primary vision technicians in Kenya given that even optometrists remain outside the public service and they play a crucial role in addressing refractive error. Therefore, it is a good suggestion but it may take longer to be implemented hence the telemedicine suggestion is ideal—Opinion leader#02

### The Kenya Society for the Blind Model

Table 5 and 6 details the SWOT analysis of the Kenya Society for the Blind Model.

**Table 5:** Strengths and weaknesses of the Kenya Society for the Blind Model.

Strengths	Weaknesses
Human resource and training	Human resource and training
Integrating individuals with skills development within the established vision centres	Only the public health facilities have integrated vision centres
Integrating telemedicine for supervision of individuals with skills development	The established scope of practise by the Ministry of Health limits the trainees from conducting refraction hence contradicting the training being undertaken
Vision centres establishment	Vision centres establishment
Establishing vision centres in public health facilities without eye care services	Lack of population based studies to guide on the rationale for establishing the vision centres
Sustainability	Sustainability
Established vision centres within different remote areas in Kenya	Short period of training on refraction
Partnership with other organization	Bulk purchase of spectacles
Quality and cost of care	Quality and cost of care
Integration of vision centres within government primary healthcare facilities	Scaling services to remote areas
Quality and affordable spectacles	Services majorly offered in urban areas
Quality and cost of care	Community engagement
A state cooperation hence highly acceptable	Not maximizing on the privileges of a government institution to scale services to the underserved population
Legal compliance	Legal compliance
Recognized and duly registered	More focus on administration with minimal focus on clinical and research

**Table 6:** Opportunities and threats of the Kenya Society for the Blind Model.

Opportunities	Threats
Engage the private sector to run the vision centres located within the public sectors	Professional discordance
Integrate the vision centres across the Public and private sectors	Lack of policies around importation of RE services such as spectacles
Prioritize reporting of URE cases from community vision screening activities	
Scale services to remote areas	

The representative from the Kenya Society for the Blind (KSB) and the team members reported that the SWOT analysis provides an insight on applicable opportunities that should be explored (quote 13).

13. Always doing a SWOT analysis provides solutions that could be adopted. I will say that the opportunities suggested such as engaging individuals from the private sector in managing the vision centres at the public hospitals is something that should be adopted—KSB representative

**Figure 3:**
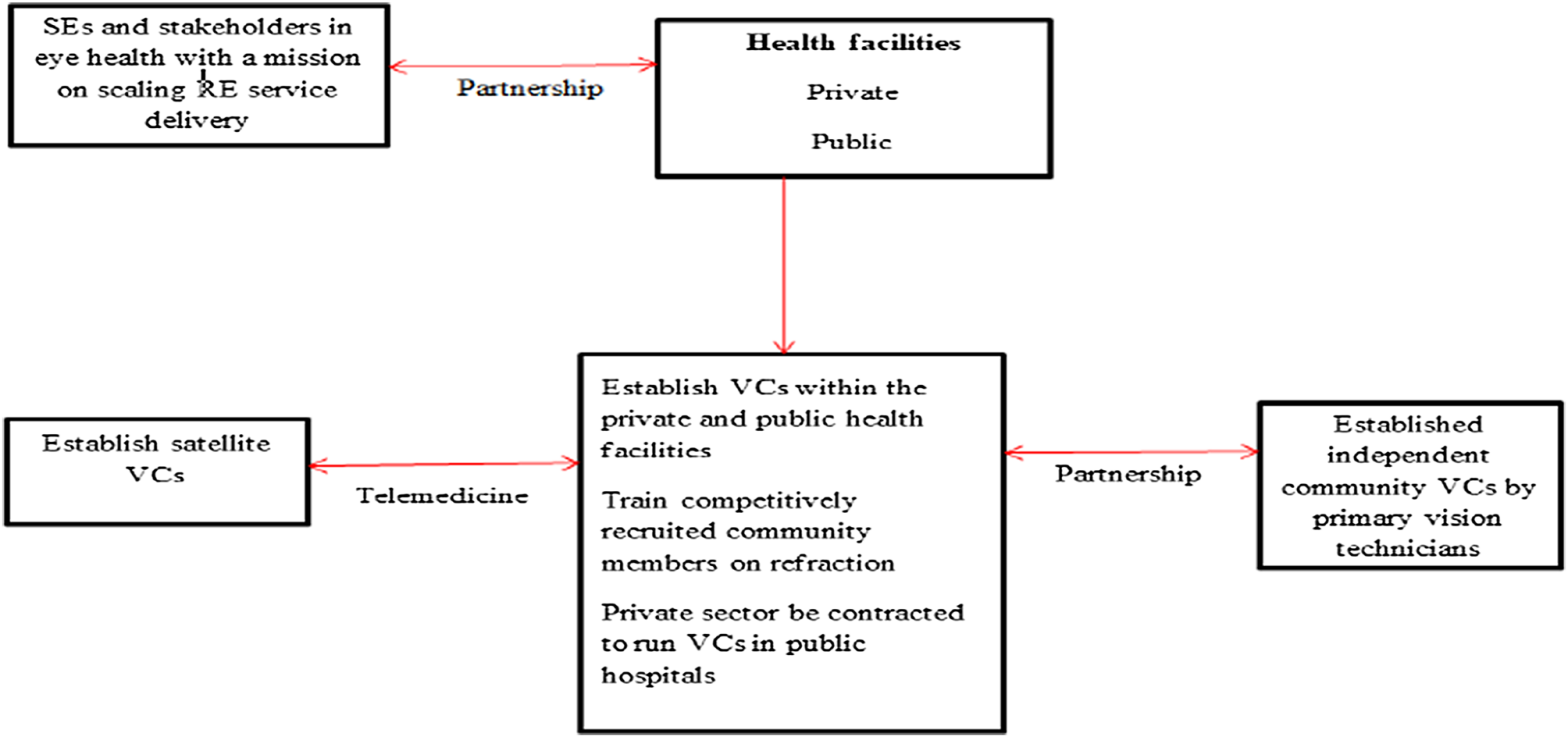
Proposed vision centre approach.

Based on the input from the KSB representatives, a proposed vision centre approach was developed as shown in Fig. 3.

Half of the key opinion leaders (n = 5; 50%) agreed that this proposed vision centre approach is appropriate and can scale effective refractive error coverage. The key opinion leaders argued that this proposed approach is inclusive and addresses aspects around human resource and service delivery in a cost effective way (quote 12). However, just under three quarters of the opinion leaders (n = 7; 70%) were neutral about the aspect of contracting the private sector to run the vision centres integrated within the public hospital sectors (quote 14).

14. In an overview of this approach, I think it can scale refractive error service delivery cost effectively given that it plans on how to scale human resource and bring in the telemedicine concept at the same time—Opinion leader#05

15. I agree the public sector has challenges which the private sectors can address, however, if we bring the private sectors then we must ensure that spectacle rates are standardized as private sector rates always remain higher and they should not bring the same trend if they are contracted to do management—Opinion leader#03

All of the key opinion leaders (100%) agreed that training of middle level healthcare professionals is a good initiative and should be prioritized (quote 16). They argued that the aspect of integrating existing primary vision technicians such as the Eye Rafikis who are already trained will be more cost effective and should be considered (quote17).

16. In most situations, it will be good if the middle level healthcare professionals can be trained on the basics of refraction because we do have limited human resource and the supervision aspect through telemedicine makes it much better and realistic—Opinion leader#06

17. I agree with the suggestion of integrating the already trained personnel’s as it will not only be cost effective and speeds up the achievement of the 2030 IN SIGHT agenda but it will enhance sustainability—Opinion leader#01

### Peek acuity

Table 7 and 8 details the SWOT analysis of the Peek Acuity.

**Table 7:** Strengths and weaknesses of the peek acuity.

Strengths	Weaknesses
Disease prevention and control	Disease prevention and control
Existing community health strategy	Weak referral pathway
Human resource and training	Retrogressive cultural beliefs and practices around eye health
Training community members on how to use the application	Human resource and training
Engage in community vision screening	Lack of awareness among the general public about the application
Established vision centres within different remote areas in Kenya	Short period of training on refraction
Awareness creation	Awareness creation
The application scales awareness	Requires a trained individual to guide in recording of visual acuity
Quality and cost of care	Quality and cost of care
Integration of vision centres within government primary healthcare facilities	Scaling services to remote areas
Cost effective for screening	Limited to recording of visual acuity
Acceptability	Acceptability
Free download with a guideline on how to utilize it	Requires a smartphone

**Table 8:** Opportunities and threats of the peek acuity.

Opportunities	Threats
Modify to allow for self-recording of visual acuity	Cost of training community members to utilize the application for screening
Integrate into an existing healthcare facility for utilization	Limited difference between a mounted visual acuity and the application
Integrate telereferral to allow trained community members to refer patients for comprehensive examination	
Design to allow the general population to self-assess and telerefer to a nearby vision centre	

The representative from the Peek Acuity and the team members reported that the SWOT analysis provides an insight on applicable opportunities that should be explored (quote 18).

18. The proposal for the modification of the application is holistic and I believe if such is undertaken then the application will not only address screening but will also enhance awareness to the general population and hospital visits—Peek Acuity representative

All of the key opinion leaders (100%) agreed that the suggestion of modifying the Peek acuity chart for utilization by everyone to self-assess will enhance awareness in a more convenient way (quote 19). The key opinion leaders denoted that if such modification can be undertaken then more RE patients will visit eye hospitals for refraction.

19. I can say this suggestion is more relevant since the big problem we do have is awareness and with the limited human resource to undertake awareness creation and service delivery concurrently, modifying the application to allow the general population to self-assess will scale not only the hospital visits but will address refractive error—Opinion leader#03

### The hub and spoke model

Table 9 and 10 details the SWOT analysis of the hub and spoke model.

**Table 9:** Strengths and weaknesses of the hub and spoke model.

Strengths	Weaknesses
Disease prevention and control	Disease prevention and control
Existing community health strategy	Weak referral pathway
Human resource and training	Limited to addressing presbyopia
Training community members on how to refract for presbyopia	Human resource and training
Engage in community vision screening	Lack of awareness among the general public about the application
Established vision centres within different remote areas in Kenya	Training 3–5 individuals to fill the role of a single vision entrepreneur
Awareness creation	Difficulty in recruiting individuals to be trained on basics of dispensing
Engage in door to door activities while dispensing reading glasses	Limiting trained individuals to only work for VisionSpring
Affordability of services	Awareness creation
Integration of vision centres within government primary healthcare facilities	Questioning on the credibility of the approach by the general public
Cost effective spectacles	Questioning on the credibility of the approach by the general public
Community engagement	Affordability of services
Targets the underserved population	Scaling services to remote areas
Sustainability	Only readers are available
Sales of subsidized reading glasses	Community engagement
	Population across the economic pyramid
	Sustainability
	Population with other condition apart from presbyopia does not benefit

**Table 10:** Opportunities and threats of the hub and spoke model.

Opportunities	Threats
· Scale to comprehensive refraction for all types of refractive errors	· Not cost effective as the trained individuals have to move from one area to the other
· Integrate telereferral to allow trained community members to refer patients for comprehensive examination	· Policy regulations
· Integrate telemedicine to allow for supervision of the trained individuals	
· Establish strategic vision centres based on population studies to allow for comprehensive refraction	
· Target everyone across the economic pyramid	
· Avail reading glasses in public and private healthcare facilities and engage in scaling awareness among the general public on the availability of the services in such facilities	
· Link the model with other existing models such as the KSB which undertakes comprehensive RE services	

The representative from the VisionSpring and the team members reported that the SWOT analysis provided was timely and identified the weaknesses of the model and possible solutions that could be adopted (quote 20).

20. This analysis provides possible solutions that could be adopted to scale refractive error services to achieve the 2030 in SIGHT in Kenya—VisionSpring representative

Given the challenge around recruitment of community members for skills development denoted within the Eye Rafiki model and the hub and spoke model, an assessment guideline was developed for competitive recruitment as shown in Table 11.

**Table 11:** Proposed recruitment assessment guideline for training primary vision technicians.

Assessment for refraction training
Demographics
Name:
Age:
Gender:
Level of education:
County:
1. What is the motivation behind your application for this refraction training?
2. Are you able to raise some finance to start a refraction point in a local setup?
3. Do you think it is possible to do business with the underserved population?
4. If yes to the above, how do you plan to raise the capital?
5. If in case you succeed to raise capital and establish a refraction point, what are some of the mechanisms you will use to influence the local population to seek your services?
6. Are you able to work as a team in case you set up a refraction point with another person who resides in your neighboring sub-location?
7. If yes to the above, which mechanisms will you apply to ensure that you work smoothly?
8. If you establish a refraction point and you realize that patients are not showing up and paying rent becomes a problem, what will you do?
9. If in case you establish a refraction point, will you work with other sectors providing similar services as yours?

All of the key opinion leaders (100%) agreed that using this assessment approach to identify community members for training will ensure that right candidates are trained. The key opinion leaders argued that using this approach will instill a sense among interested community members that there is a burden called refractive error that they are destined to work towards addressing (quote 21).

21. For me I will say one of the challenges we have is recruitment of community members tasked with provision of refractive error services in Kenya. As a result I think using something like the suggested will ensure we get right candidates and the process remain competitive hence creation of commitment by the trained individuals—Opinion leader#07

All of the key opinion leaders (100%) agreed that the human resource remains a major challenge in the eye health ecosystem in Kenya and a structured approach should be designed to ensure that anyone trained have the potential to deliver RE services contributes towards enhancing effective RE coverage (quotes 22).

22. Mostly it is hard for the trained community members to deliver refractive error services effectively since what they do remains unknown among other eye care professionals hence through a structured approach, all the trained personnel’s can be distributed to scale effective refractive error—Opinion leader#01

**Figure 4:**
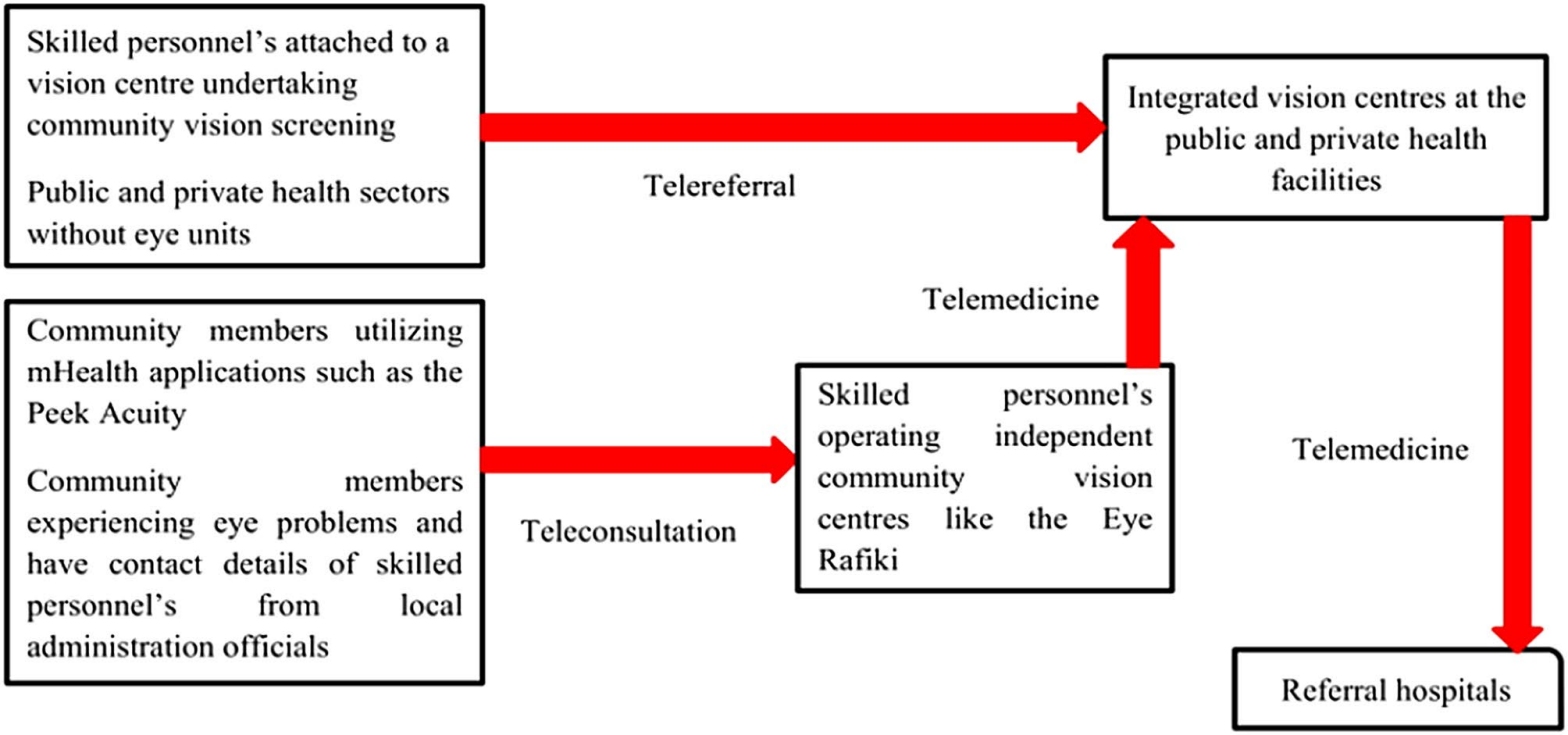
Proposed referral approach.

Addressing the weak referral pathway was proposed as shown in Fig. 4.

All of the key opinion leaders (100%) agreed that the proposed referral approach is appropriate as it integrate the concept of telemedicine and gives the primary vision technicians a role to play within the referral pathway. The key opinion leaders argued that this referral approach is inclusive of all players within the eye health ecosystem (quotes 23–24).

23. Telemedicine would be a more suitable tool in addressing refractive error in Kenya given that the human resource remains limited and most optical shops are in urban areas—Opinion leader#03

24. I only have to say that this approach will ensure quality refractive error service delivery and a more holistic approach in addressing refractive error since cases beyond the scope of any cadre will be referred easily—Opinion leader#07

### Operation eyesight universal model

Table 12 and 13 details the SWOT analysis of the Operation Eyesight Universal model.

**Table 12:** Strengths and weaknesses of the operation eyesight universal.

Strengths	Weaknesses
Vision centres establishment	Vision centres establishment
Establish eye units within the public hospitals	Exempting the private hospitals
Human resource and training	Establishment of eye units in public hospitals not based on evidence from population based studies
Training of teachers to do vision screening using peek acuity application	Absence of spectacles within the established eye units
Support community vision screening	Human resource and training
Established vision centres within different remote areas in Kenya	Lack of telemedicine integration between teachers and eye care professionals
Awareness creation	Weak referral pathway
Application of the Peek Acuity to undertake screening	Awareness creation
	Minimal engagement of existing eye care professionals

**Table 13:** Opportunities and threats of the operation eyesight universal.

Opportunities	Threats
· Introduce subsidized spectacles within the established eye units so as to generate income for sustainability	· Resistance from the dominant commercial enterprise sectors engaged in the optical business
· Integrate telereferral to allow trained teachers to refer patients for comprehensive examination	
· Integrate telemedicine to allow for supervision of the trained teachers	
· Establish eye units based on population studies to allow the population in need of RE services benefits from the services	
· Engage the private sectors	

The representative from the Operation Eyesight Universal and the members reported that the proposed opportunities could potentially scale RE services if adopted (quote 25).

25. Always generating income for sustainability of the model is key as we fulfill our social mission. Hence the proposed opportunities are desirable and the weaknesses should be addressed—Operation Eyesight Universal representative

All of the key opinion leaders (100%) agreed that the aspect of training the teachers to do vision screening for pupils is an important aspect as it will ensure quality of life and academic performance among the pupils. They argued that integrating telemedicine will ensure that the trained teachers manage RE appropriately and refer cases beyond their scope (quotes 26–27).

26. Training teachers to undertake vision screening is a cost effective way that will ensure that refractive error status for pupils is diagnosed early allowing effective refractive error management—Opinion leader#05

27. I agree with the telemedicine integration because it will ensure quality delivery given that the teachers are given basic training and if they are supervised through telemedicine then the 2030 IN SIGHT will be achieved—Opinion leader#08

### Fred hollows foundation approach

Table 14 and 15 details the SWOT analysis of the Fred hollows foundation approach.

**Table 14:** Strengths and weaknesses of the Fred Hollows Foundation.

Strengths	Weaknesses
Disease prevention and control	Disease prevention and control
Advocates for integration of eye health services into the national and county health strategic plans	Limited advocacy towards integration of eye health services into the private sectors
Human resource and training	Limited support for population based studies
Sponsor training of ophthalmologists	Human resource and training
Sponsor training of middle level healthcare professionals	Minimal attention towards addressing URE as training of optometrist’s remains a non-priority
Support vision screening activities	Weak referral pathway
Availability of RE services	Availability of RE services
Support existing public eye units in Kenya	Lack of RE services such as spectacles within the supported eye units
Affordability of services	Affordability of services
Integration of vision centres within government primary healthcare facilities	Lack of prescription lenses for myopes and hyperopes

**Table 15:** Opportunities and threats of the Fred Hollows Foundation.

Opportunities	Threats
· Introduce subsidized spectacles within the established eye units and generate revenue for sustainability	· Excluding optometrists in their activities
· Engage optometrists when it comes to URE	· Not addressing URE and cataract concurrently
· Integrate telemedicine to ensure that the middle level healthcare professionals trained can deliver RE services under supervision of an optometrist	
· Establish eye units based on population studies to allow the population in need of RE services benefits from the services	
· Equip the trained community health volunteers with mHealth applications like the Peek Acuity so as to undertake community vision screenings	
· Engage the private sectors	

The representative from the Fred Hollows Foundation (FHF) reported that advocating for integration of subsidized spectacles within the public health sector is desirable (quote 28).

28. Since correction of RE simply require a simple pair of spectacles, the suggestion of integrating subsidized spectacles within the public health sectors is worthy of attention-FHF representative

All of the key opinion leaders (100%) agreed that equipping community health volunteers with screening tools like the PA mobile acuity will scale vision screening and effective RE coverage. They argued that integrating the optical aspects in the government health facilities is relevant (quotes 29).

29. I think if this suggested approach can be implemented then refractive error service delivery will be scaled since the big challenge we have is lack of refraction points and if such can be created and be coordinated by telemedicine then the 2030 IN SIGHT will be achieved—Opinion leader#01

### DOT glasses approach

Table 16 and 17 details the SWOT analysis of the DOT glasses approach.

**Table 16:** Strengths and weaknesses of the Dot glasses.

Strengths	Weaknesses
Disease prevention and control	Disease prevention and control
Door to door screening	Other forms of RE such as myopia and hyperopia remains unaddressed
Equipping existing human resource in eye health with vision kits to undertake community activities	More concentration in urban areas
Human resource and training	Only readers are available
Training of entrepreneurs to undertake sales of reading glasses	Human resource and training
The trainees are provided with complete kits with vision testers and a selection of lenses	Limited skilled human resource
Support vision screening activities	Weak referral pathway
Sustainability	Sustainability
Sales of subsidized reading glasses	Limited from selling prescription lenses

**Table 17:** Opportunities and threats of the DOT glasses.

Opportunities	Threats
· Introduce subsidized spectacles within the established eye units and integrate the trained individuals to the established eye units	· Questions around the credibility of the trained personnel’s
· Equip a public or private health facility in areas with dire need of RE services and introduce subsidized spectacles	· Limited from achieving the 2030 in SIGHT as only presbyopia is being addressed
· Integrate telemedicine to ensure that the middle level healthcare professionals trained can deliver RE services under supervision of an optometrist	
· Integrate mHealth applications such as the Peek Acuity to scale screening activities	
· The model can be linked with the KSB model which undertakes comprehensive RE management	

The representative from DOT Glasses and the team members reported that the proposed opportunity around integration of the individuals they train to existing eye units in the public and private sector could potentially scale sustainability (quote 30).

30. Normally the individuals we train do operate independently and as a result they experience various challenges. Hence the proposed approach for integration into the existing public and private health sectors could enhance sustainability as they will be selling subsidized spectacles—DOT Glasses representative

All of the key opinion leaders (100%) agreed that the suggestion for linking the Dot Glasses approach with the KSB model can address refractive error effectively. The key opinion leaders argued that because one model addresses only presbyopia while the other addresses all aspects of RE, the linkage will supplement the model to ensure comprehensive RE (quote 24).

31. Clearly if this proposed approach can be utilized then it will ensure the cases seen at Dot Glasses who require prescription spectacles can be sent to the KSB model for dispensing—Opinion leader#10

All of the key opinion leaders (100%) agreed that integrating telemedicine an embracing a skills development for primary vision technicians is worthy of attention. The opinion leaders denoted that the efforts directed toward scaling human resource in eye health is crucial and the aspect of supervising their activities through telemedicine will make the primary vision technicians more relevant in the eye health ecosystem (quotes 32–33).

32. One of the most critical suggestion with this approach is that it will make the primary vision technicians more useful in the delivery of refractive error services given that they will be getting supervision from qualified eye care professionals like optometrists—Opinion leader#03

33. I agree with the suggestion on telemedicine integration and the mHealth applications because it will ensure that the primary vision technicians can carry screening activities effectively through the Mhealth applications and get supervision through telemedicine—Opinion leader#07

## Discussion

The current national strategic plan for eye health in Kenya^13^ incorporates RE in its plan but has not shown how they intend to achieve the targets holistically given the limited resources available. This could be attributed to the fact that the prevalence of URE in Kenya from population based studies remains unknown^27^ resulting in minimal efforts directed towards addressing URE. As a result, this review denotes the need for establishment of a national strategic plan inclined towards addressing URE as shown in Fig. 1 to act a guideline for the current models used by SEs in Kenya. However, for such national strategic plans to be established, optometrists in partnership with all stakeholders in eye health should be at the forefront in undertaking population based studies to showcase to the government the need for prioritization of URE. With the devolved system of governance in Kenya^28^, addressing URE may only be achieved if each devolved unit direct efforts towards addressing URE. Hence it is anticipated that the proposed strategic plan may facilitate a balanced establishment of refraction points and distribution of human resource in different geographical locations as opposed to the current situation where most optical units are located within urban areas^27^. Notwithstanding, it is also anticipated that establishing a strategic plan will ensure that RE indicators are integrated within the hospital information system for easy tracking through a quality assurance team. Therefore, the current models used by SEs should engage in research and periodically collect and report on effective RE coverage so as to achieve the 2030 in SIGHT.

Human resource remains a major challenge in the eye health ecosystem in most developing countries such as Kenya^27^. Being that human resource is a critical aspect necessary for the achievement of the 2030 IN SIGHT, the conventional training approach needs to be supplemented with other cost effective approaches. This SWOT analysis has showed that only the KSB model does not undertake skills development. This could be attributed to the fact that the KSB is a state corporation hence not dynamic when compared to other SEs. This study has also showed that some SEs such as the Peek Acuity, KSB and Operation Eyesight Universal have embraced technology integration into their activities. Given that access to RE services has been a major challenge among the underserved population, evidence shows that application of telehealth in primary eye care reduces logistical barriers faced by vulnerable patients^29^. According to the World Health Organization, four out of five developing nations now offer at least one type of mobile health program to deliver essential health services to the population^30^. A meeting abstract by Mair and Whitten^31^, examining telemedicine delivered subjective refraction found no statistically significant difference between in-person and telemedicine delivered subjective refraction. Therefore, to achieve the 2030 in SIGHT in Kenya, the existing SEs models should embrace integration of technology such as telemedicine so as to strengthen trained individuals through skills development and ensure quality RE service delivery.

Social enterprises attempts to address social needs not addressed by the government and or the commercial enterprise sector^6^. In Kenya, most eye care professionals operate from urban areas as most optical units are situated in urban areas hence limiting the underserved from rural areas from accessing RE services^27^. A World Bank, survey revealed that social enterprises depend mostly on donations and subsidy of services for sustainability^32^. However, to achieve the 2030 in SIGHT in Kenya, sustainability of the models should be prioritized. This study result has showed that apart from the FHF, Operation Eyesight Universal and Peek Acuity, all the other SEs models apply approaches such as sales of subsidized spectacles to ensure sustainability. This is contrary to the LV Prasad Eye institute which declined donor funding and funds from other organizations with repayment terms and conditions^33^. Given that SEs addresses inequality more broadly by acting on the social, economic and environmental circumstances of the most vulnerable members of the society, factors such as financial constraints and policy regulations influences sustainability^34^. This study has identified policy regulation and conflict of interest among eye care professionals and individuals trained by SEs which influence their sustainability. Therefore, a suitable ecosystem should be provided for SEs to ensure an efficient delivery of RE services.

Being that approximately 90% of the populations in developing countries are in need of RE services^35–37^, awareness creation is a key important aspect desirable for the achievement of the 2030 IN SIGHT. This study has shown that some models are enhancing awareness creation on RE in Kenya. This could be attributed to the current situation in which the awareness around RE services has received minimal attention by the government hence SEs work towards bridging this gap. Although awareness creation among the general population is an important aspects^38^, the population at the base of economic pyramid may not seek the services if awareness is not enhanced. Again this study has showed that community engagement is a key aspect considered by some SEs models currently delivering RE services in Kenya. Evidence shows that almost half of the world population will suffer from myopia by 2050 warranting the need for a strengthened community engagement to curb the burden^39^. As a result, strengthening awareness creation and community engagement is desirable to enhance achievement of the 2030 IN SIGHT.

Accessibility, availability and affordability remain the key barriers influencing RE service delivery in Sub-Saharan Africa^40^. As a result, the public sectors are trying to address the barriers around accessibility, availability and affordability of RE services. However, given the limited resources available in developing countries^27^, other stakeholders in eye health such as the SE are trying to supplement the efforts of the public sectors by deploying cost effective models targeting the population across the economic pyramid so as to achieve the 2030 IN SIGHT. The current study findings acknowledge that with some models, SE in Kenya are establishing refraction points with cost effective spectacles within rural areas to enhance accessibility, availability and affordability. This initiative if acknowledged and supported by the government has the potential to propel the achievement of the 2030 IN SIGHT in Kenya since continuity in RE service delivery demands sustainable financing approaches. Non-affordability and poor accessibility of the services among rural areas are identified as an important indication for the high prevalence of blindness^41^. However, given that RE services are not available in many public health sectors in Kenya^27^, the models used by SE should advocate for a partnership with the public health sector so that they can introduce RE services within the public health sector and adopt a team approach to aid in the achievement of the 2030 IN SIGHT in Kenya.

Most SEs have proven to be innovative as per the models they are using to deliver URE in Kenya across the economic pyramid in Kenya. However, a review of the current models used by SE in Kenya suggests that they can only achieve the 2030 IN SIGHT if a strong partnership is enhanced amongst them. The findings of this study has also noted that the current models used by SE in Kenya remains weak in terms of referral and replication of concepts amongst the social enterprises. The weak referral could be attributed to absence of a strengthened relationship between the SE and the public sectors. Although the social entrepreneurship concept is taking shape in Kenya^42^, existing SE face challenges which limit their full potential when it comes to RE service delivery. As a result, the government should provide a supportive environment for SE so as to undertake activities leading to achievement of the 2030 IN SIGHT agenda in Kenya. A team approach and partnership is worthy of attention across all sectors in eye health so as to facilitate the 2030 IN SIGHT achievement in Kenya. Therefore, with some models of SE in Kenya utilizing technology such as the Peek Acuity^43^, replication of some concepts is desirable to scale RE service delivery and achieve 2030 IN SIGHT agenda.

In conclusion, with the dynamic nature of the models used by SE, they are well placed to propel the achievement of the 2030 IN SIGHT in Kenya. Therefore, to achieve the 2030 in SIGHT in Kenya, the current models used by SE in Kenya should explore the proposed opportunities from the SWOT analysis. Some of the key opportunities that should be prioritized include integration of telemedicine, community engagement, awareness creation, advocacy towards policy changes and sustainability.

## Data Availability

The datasets used and/or analyzed during the current study available from the corresponding author on reasonable request.

## Abbreviations

RE: Refractive error
SE: Social enterprise
URE: Uncorrected refractive error
